# Prognostic value of some tumor markers in unresectable stage IV oropharyngeal carcinoma patients treated with concomitant radiochemotherapy

**DOI:** 10.2478/raon-2014-0048

**Published:** 2015-11-27

**Authors:** Erika Soba, Marjan Budihna, Lojze Smid, Nina Gale, Hotimir Lesnicar, Branko Zakotnik, Primoz Strojan

**Affiliations:** 1 Institute of Oncology Ljubljana, Ljubljana, Slovenia; 2 University Department of Otorhinolaryngology and Cervicofacial Surgery, University Clinical Center, Ljubljana, Slovenia; 3 Institute of Pathology, Faculty of Medicine, University of Ljubljana, Ljubljana, Slovenia

**Keywords:** oropharynx, radiochemotherapy, tumor markers

## Abstract

**Background:**

The aim of the study was to investigate how the expression of tumor markers p21, p27, p53, cyclin D1, EGFR, Ki-67, and CD31 influenced the outcome of advanced inoperable oropharyngeal carcinoma patients, treated with concomitant radiochemotherapy.

**Patients and methods.:**

The pretreatment biopsy specimens of 74 consecutive patients with inoperable stage IV oropharyngeal squamous cell carcinoma treated with concomitant radiochemotherapy were in retrospective study processed by immunochemistry for p21, p27, p53, cyclin D1, EGFR, Ki-67, and CD31. Disease-free survival (DFS) was assessed according to the expression of tumor markers.

**Results:**

Patients with a high expression of p21 (≥10%), p27 (>50%), Ki-67 (>50%), CD31 (>130 vessels/mm2) and low expression of p53 (<10%), cyclin D1 (<10%) and EGFR (<10%) (favorable levels - FL) had better DFS than patients with a low expression of p21 (<10%), p27 (≤50%), Ki-67 (≤50%), CD31 (<130 vessels/mm2) and high expression of p53 (≥10%), cyclin D1 (≥10%) and EGFR (≥10%) (unfavorable levels - UL). However, statistical significance in survival between FL and UL was achieved only for p27 and cyclin D1. DFS significantly decreased with an increasing number of markers with an unfavorable level per tumor (1–4 vs. 5–7) (78% vs. 32%, respectively; p = 0.004). The number of markers per tumor with UL of expression retained prognostic significance also in multivariate analysis.

**Conclusions:**

Statistical significance in survival between FL and UL emerged only for p27 and cyclin D1. The number of markers per tumor with UL of expression was an independent prognostic factor for an adverse outcome.

## Introduction

Prognostic evaluation of patients with unresectable squamous cell carcinoma in the head and neck (SCCHN) is currently based on the performance status of the patient and the tumor stage. Recently, the presence of human papillomavirus (HPV) DNA in tumor cells has also been identified as a strong predictor of survival in patients with oropharyngeal primaries.^1^ However, these classical prognostic factors often do not provide sufficient information for the selection of the optimal therapy. The changes in the genes or their products which can be traced biochemically or immunohistochemically may serve to estimate the aggressiveness of the tumor and, consequently, to adapt treatment accordingly.

On the basis of the favorable results of our prospective randomized clinical study^2^, concomitant radiochemotherapy with mitomycin C and bleomycin was introduced in the 1990s as a routine treatment for patients with unresectable squamous cell carcinoma of the oropharynx (SCCOP) in our department. Because the treatment morbidity was rather severe also in those patients who were not cured^3^, the identification of patients from a homogenous group (in regard to the primary tumor origin, stage and treatment), who will or will not respond to aggressive radiochemotherapy would help in sorting patients into various treatment programs of different intensity, to spare some of them from unnecessary toxicity. Therefore, the primary aim of our study was to investigate how the expression of growth promoting (cyclin D1, epidermal growth factor receptor [EGFR], Ki-67) and growth suppressing (p21, p27, p53) tumor markers and CD31 in the primary tumor tissue influenced the outcome of patients with unresectable SCCOP.

## Patients and methods

### Patients

The 95 consecutive patients with previously untreated, technically inoperable SCCOP were treated with curative intent in the period 1991–1998 with irradiation and concomitant application of mitomycin C and bleomycin. To achieve maximal possible homogeneity of the studied group, 21 patients with disease of UICC TNM stage III were excluded for further evaluation. Patients were irradiated five times weekly with one fraction of 2 Gy/day with a planned total dose of 66–70 Gy. The physical dose was converted into a biologically effective dose (BED) according to the formula: BED = TD × (1 + d/α/β) – K × (Tt – Td), where TD = total tumor dose in Gy, Tt = total treatment time in days, K = daily dose equivalent of repopulation in units of Gy_α/β_ per day (K = 0.6, and α/β = 10), and Td = lag time in days to the onset of effective repopulation during the treatment (4). It was assumed that Td = 28 days.

The chemotherapy regimen consisted of intramuscular applications of bleomycin 5 mg twice a week with the planned dose being 70 mg and one application of mitomycin C 15 mg/m^2^ applied intravenously after delivery of 9–10 Gy of irradiation. Radiotherapy was considered intensive if the BED was ≥65Gy10. Chemotherapy was considered intensive if the dose of mitomycin C was ≥14.1 mg/m2 and of bleomycin ≥35mg. The whole treatment was considered intensive, if chemotherapy or radiotherapy or both were intensive.

## Methods

### Immunohistochemistry

The pretreatment biopsy specimens of the primary tumors were in retrospective clinical study processed by immunochemistry for p21, p27, p53, Cyclin D1, EGFR, Ki-67, and CD31. Immunohistochemistry was performed on 4 μm paraffin sections mounted on silicon-coated glass slides. The antibodies and preconditioning applied for immunohistochemistry are presented in Table 1.

To determine the level of expression of the tested markers, semi-quantitative scoring of immune reactivity was performed according to the percentage of positivity in the tumor cells as follows: 0 = less than 10%, 1 = 10–50%, 2 = more than 50% of tumor cells with a positive reaction (for p21, p27, p53, Ki-67, cyclin D1 and EGFR). Microvascular density (MVD) was assessed quantitatively with the CD31 antibody. Stained microvessels were counted and expressed as the number of microvessels per mm^2^ in the areas of maximal neovascularization of the tumor stroma.

### Statistical methods

Disease free survival (DFS) was defined as the time interval from the beginning of the treatment to the appearance of local and/or regional progression and/or distant metastases. The survival curves were plotted by using the method of Kaplan-Meier^5^ and a log rank test was used to test the differences in survival between subgroups.^6^ Survival was calculated in subgroups defined by the level of expression of tumor markers, performance status (PS) and intensity of treatment. For multivariate analysis, a Cox proportional hazards model was used.^7^

### Ethical consideration

The study was carried out according to the Helsinki Declaration (1964, with later amendments) and of the European Council Convention on Protection of Human Rights in Bio-Medicine (Oviedo 1997). It was approved by the Institutional Review Board Committee and by the National Committee for Medical Ethics, Ministry of Health, Republic of Slovenia

## Results

Because of severe mucositis, some patients could not be irradiated to the planned dose or receive the full dose of bleomycin. Some patients had mild liver damage and the dose of mitomycin had to be below the planned dose. So, the intensity of the treatment was not the same for all patients.

Only in 59 out of 74 patients with UICC stage IV of SCCOP was there sufficient biopsy tissue in paraffin blocks for the analysis of all seven tumor markers under investigation. Among them, there were 2 females and 57 males with a median age of 52 years (39–67 years). In 42 patients the WHO PS was 0, and in 17 it was assessed as 1–2. The treatment intensity was low in 13 patients and high in 46.

The distribution of patients according to the expression of markers in the tumor tissue is presented in Table 2

The median follow-up time of patients was 6 years (3–10 years). Twenty nine (49%) patients had a local, regional or distant relapse; at the last follow-up 15 (25%) were still alive and 44 (75%) were dead. The probability for DFS at five years was 48%. Intensive treatment (high vs. low) and good PS (0 vs. ≥1) were associated with statistically significantly better DFS: 59% vs. 9%, p = 0.000; 58% vs. 19%, p = 0.005, respectively. In the subgroup of intensively treated patients and good PS, DFS was 65 %, while for those with a poor performance status it was 30% (p = 0.08).

**FIGURE 1.**
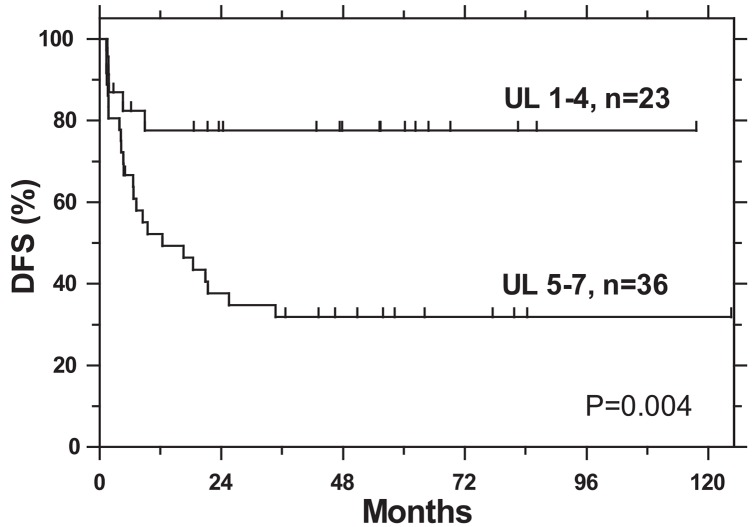
The disease-free survival (DFS) of patients according to the number of markers with unfavorable level of expression per tumor (UL).

Patients with a favorable expression profile (FL) of studied markers, *i.e.* a high expression of p21 (≥ 10%), p27 (> 50%), Ki-67 (> 50%), CD31 (> 130 vessels/mm^2^) and low expression of p53 (< 10%), cyclin D1 (< 10%) and EGFR (< 10%), had better DFS than patients with an unfavorable expression of these markers, *i.e.* p21 (< 10%), p27 (≤ 50%), Ki-67 (≤ 50%), CD31 (< 130 vessels/mm^2^) and a high expression of p53 (≥ 10%), cyclinD1 (≥ 10%) and EGFR (≥ 10%). However, statistical significance in DFS between FL and UL was achieved only in the case of p27 and cyclin D1. DFS significantly decreased with an increasing number of markers with UL per tumor (1–4 vs. 5–7): 78% vs. 32%, p = 0.004 (Table 3, Figure 1). Considering the expression of only p27 and cyclin D1, apart from other markers, DFS was significantly worse for those patients whose tumor had unfavorable expression levels of both markers: (0–1 vs. 2): 65% vs. 17%, respectively (p = 0,002) (Table. 3). Intensively treated patients in poor PS with FL of expression of p27 survived significantly better than those with a low expression of p27 (75% vs. 0%, p = 0.017). In the case of cyclin D1, corresponding analysis was not possible due to a small number of intensively treated patients with FL of this marker.

In a Cox proportional regression model, PS, intensity of treatment and p27 retained statistical significance (HR = 2.363, 95% CI = 0.999–5.589, p = 0.050; HR = 2.550, 95% CI = 1.105–5.886, p = 0.028; HR = 3.743, 95% CI =1.064–13.169, p = 0.040, respectively) while cyclin D1 was marginally significant (HR = 1.070, 95% CI = 1.000–1.145, p = 0.051). Compared to p27, the number of all markers per tumor with UL of expression had a stronger statistically significant influence on the prognosis (HR = 3.614, p = 0.009), along with the intensity of treatment (HR = 3.150, p = 0.005) and PS (HR = 2.352, p = 0.031).

## Discussion

Information on the prognostic value of different tumor markers in patients with unresectable SCCOP, treated with concomitant radiochemotherapy is scarce. Out of several known tumor markers we choose some growth promoting (cyclin D1, EGFR, Ki-67) and growth suppressing (p21, p27, p53) markers, and CD31. In addition to PS, which is one of the stronger prognostic factor in different malignant diseases^8^, and treatment intensity, only the number of markers with FL of expression in a particular tumor and p27 (as an individual marker) were recognized as independent prognosticators for DFS.

The published results on the value of these tumor markers in SCCHN vary. For example, in the case of p27, a negative cell-cycle regulator that blocks progression from late G1 to S phase^9^, its protein expression was found to positively correlate with disease outcome in some studies^9^–^12^, although a negative relationship was also described.^13^ Probably the most widely studied marker is the nuclear transcription factor p53, playing a role in the control of cell proliferation, apoptosis and maintenance of the fidelity of DNA duplication.^14^,^15^ The results of a meta-analysis on the role of upregulated p53 in patients with SCCHN were inconclusive, mainly due to large heterogeneity across the studies.^16^ Interestingly, in three rather homogeneous studies with SCCOP^17^–^19^, meta-analysis indicates that p53 overexpression/mutation confers a survival advantage.^16^ Discrepancies in the results of different studies can also be found in the case of p21 (G1-phase blocker)^20^–^23^, cyclin D1 (promotes progression of cells throughout the cycle)^24^–^26^, Ki-67 (a measure of the proliferative capacity of the tumor)^27^–^29^, EGFR (cell growth promoter)^30^,^31^, and CD31 (microvascular density indicator).^32^–^34^ In our study, the HPV testing was not performed. However, because 90% of the patients from our series were heavy smokers (*i.e.* with lifetime tobacco exposure of one pack of cigarettes per day for ≥10 years) - a fact that negatively influences immune system activity, which is crucial for the favorable outcome observed in HPV-positive tumors - the tumor HPV status in our patients would be less likely to play a significant role.^35^,^36^

Survival of our patients, who represent a rather homogeneous group regarding histology, primary tumor localization, stage, and treatment, depended primarily on the intensity of the applied therapies, their PS and also on the biological characteristics of the tumor. The latter was determined by studying dysregulation in the expression of seven tumor markers, and was influenced also by several other pathologic processes taking place in the tumor, not considered in our study. It was found that a low expression of p21, p27, Ki-67, CD31 and high expression of p53, cyclin D1 and EGFR negatively influenced DFS. In general, when analyzing separately the expression of each of the seven markers, the difference in their expression showed no statistically significant correlation with survival probability; the two exceptions were p27 and cyclin D1. In addition to the lack of prognostic potential as an intrinsic characteristic of an individual marker, another reason for negative results could be the low number of patients in our series. However, the influence of studied markers on survival increased above the level of statistical significance, when the sum of only those markers with UL per tumor was taken into account (Figure1). By ranking the patients according to this criterion, we found a significantly lower DFS in the group with the increased number of markers with UL of expression. In the multivariate analysis, the sum of UL of expression of markers per tumor remained an independent prognostic factor for DFS, along with PS and intensity of treatment. It appears that the prediction of the outcome of the disease on the basis of expression of only one marker, even in a homogeneous group of patients, is not necessarily successful. The expression profiles of different genes are interdependent and none of the known tumor markers can play independently inside this network. Accordingly, the expression level of a particular maker resulted from the sum of influences exerted by a variety of other markers and vice versa. This might also explain why the opinions on the prognostic value of individual markers in the literature differ to such a great extent.

As expected, the survival of our patients with poor PS was low. Among them we tried to identify those who did not benefit from rather toxic concomitant radiochemotherapy. Intensively treated poor PS patients with ≤ 4 UL markers and those who had FL of expression of one or both of p27 and cyclin D1 had comparable DFS (67% and 60% respectively) to intensively treated patients with good PS. On the other hand, in spite of intensive treatment, patients with poor PS with > 4 UL markers and those with UL of expression of one or both of p27 and cyclin D1 had poor DFS (17% and 0% respectively). It seems that these patients should not be treated that vigorously and are candidates for palliative treatment programs.

In conclusion, in a series of 59 SCCOP patients, uniformly treated with concomitant radiochemotherapy with mitomycin C and bleomycin, a set of seven markers, determined immunohistochemically, was recognized as a significant predictor of DFS only when the number of markers per tumor with UL of expression was considered. Of the individual markers, only p27 correlated with survival on multivariate analysis, in addition to the PS of the patients and the intensity of the applied therapies. Despite intensive treatment, poor PS patients with > 4 markers with UL of expression as well as those with UL of expression of p27 and cyclin D1, had unfavorable survival rates: these patients should be treated with palliative intent.

## Figures and Tables

**TABLE 1.** Antibodies and preconditioning applied for immunohistochemistry

**Antibody**	**Clone**	**Producer**	**Dilution**	**Preconditioning**
**p21**	SX118	DAKO	1:10	MW*, 6 min, EDTA buffer, pH 8.0 cooling 10 min
**p27**	1B4	Novocastra	1:10	MW*, 6 min, EDTA buffer, pH 8.0 cooling 10 min
**p53**	DO7	DAKO	1:50	MW*, 6 min, EDTA buffer, pH 8.0 cooling 10 min
**Ciklin D1**	P2D11F11	Novocastra	1:10	MW*, 6 min, EDTA buffer, pH 8.0 cooling 10 min
**EGFR**	H11	DAKO	1:10	Proteinase 1 (Ventana) 12 min
**Ki-67**	MIB1	DAKO	1:20	MW*, 6 min, EDTA buffer, pH 8.0 cooling 10 min
**CD31**	JC/70A	DAKO	1:15	MW**, 7 min, 96°C, citr. buffer, pH 6.0 + Proteinase 1 (Ventana), 2 min

EGFR = epidermal growth factor receptor; EDTA = ethylene-diamine-tetraacetic acid;

MW^*^ = common microwave oven;

MW^**^ = microwave oven Polar Patent

**TABLE 2.** The distribution of patients according to the expression of tumor markers

**Intensity of expression**	**P21**	**P27**	**P53**	**Cyclin D1**	**EGFR**	**Ki-67**	**CD31**
**< 10%**	20	22	28	31	13	13	Microvascular density in stroma: Median 118 (48–206) per mm^2^
**10%–50%**	26	23	9	21	15	33	< 130 (n = 40)
**> 50%**	13	14	22	7	31	13	> 130 (n = 19)
**Total**	59	59	59	59	59	59	59

EGFR = epidermal growth factor receptor

**TABLE 3.** Disease-free survival at 5 years according to the expression of tumor markers

**Marker**		**Expression**	**n**	**DFS %**	**p**
p21	UL	< 10%	20	34	0.204
	FL	≥ 10%	39	55	
p27	UL	≤ 50%	45	40	0.040
	FL	> 50%	14	77	
p53	FL	< 10%	28	59	0.177
	UL	≥ 10%	31	38	
Cyclin D1	FL	< 10%	31	66	0.020
	UL	≥ 10%	28	30	
EGFR	FL	< 10%	13	77	0.093
	UL	≥ 10%	46	41	
Ki-67	UL	≤ 50%	46	42	0.131
	FL	> 50%	13	68	
CD31^*^	UL	< 130	40	40	0.100
	FL	> 130	19	69	
Number of UL per patient 1–4 vs 5–7		1–4	23	78	0.004
		5–7	36	32	
Number of UL only for P27 & cyclin D1		0–1	39	65	0.002
		2	20	17	

DFS = disease free survival; UL = unfavorable level of expression of tumor marker; FL = favorable level of expression of tumor marker; EGFR = pidermal growth factor receptor; n = number of patients;

* in micro-vessels per mm^2^.
